# Sensitive Dentine

**Published:** 1872-12

**Authors:** M. M’Carty


### Sensitive Dentine.

BY M. M'CARTY.

*Read before the Tennessee State Dental Association.*

According to my preconceived views sensitive dentine is a misnomer. It is a law of nature that no two substances can occupy the same space at the same time.

The dentine is composed of lime salts, which form the tubes in which are incased the nerve fibrils that are always normally sensitive in a sound, healthy tooth; but under certain circumstances, hereinafter to be mentioned, are susceptible of taking on exalted sensibility. The dentinal nerve fibrils possessing sensation are located in these tubes of dentine and enamel of the teeth. Now we cannot cut the dentine without doing injury to the dentinal nerve fibrils; pain is felt, and we are taught to call it "sensitive dentine."

Our experience has settled the fact in our mind that the nerve fibrils may be destroyed in various ways; and yet the dentine remain intact without any sensation whatever. For instance, when we devitalize the pulp of a tooth with arsenious acid all the sensibility departs with the life of the pulp, because the dentinal branches come from the pulp. And I infer that in case of the severance of the trunk or branch of a nerve leading to a tooth, that the tooth would be left without sensibility in the dentine and enamel. The cementum receives its nerves from the dentinal periosteum.

Hence, I conclude that the dentine is not sensitive, but the nerve fibrils situated therein; for the dentine is one substance occupying one space, while the nerve fibrils is another substance occupying another space. And we have already shown that one (the nerve fibrils) can be devitalized without destroying the integrity of the other. But as it is known as "sensitive dentine," I accept the term, and will write of it as such.

In the human system we have three kinds of nerves:—viz: nerves of common sensation; nerves of special sense; and nerves of motion. Some nerves seem to perform two of these functions. They are called mixed nerves. Of the first we will only have to write, as we find no other nerve in the teeth, except that of common sensation.

Our Maker, when he made man, gave him nerves of common sensation for a wise and beneficent purpose. And he placed them in our teeth for the same reason that they are distributed to all the other organs of our system, that we may feel a sense of comfort, pleasure, yea delight, when in a healthy condition, and a sense of pain when injury is being inflicted.

No matter what the character of injury is that is, being received by any organ or organs, nor the cause of it, the nerves of common sensation stand as sentinels, and always act as willing and prompt monitors, giving us notice to take care of the part or parts, or the whole that is receiving the injury. How essential they are then to our existence. What a necessary provision this was in our Creator. For without this knowledge of common sensation, its constant warnings, we would not be capable of taking care of ourselves for a day.

Passing on we will proceed to consider the subject assigned us more minutely. It is known as exalted sensibility of the dentine, inflamed dentine, and sensitive dentine. What is it? It is a pathological condition in a tooth having resulted from a lesion of continuity in the tooth. This pathological condition may be a sequence of mechanical abrasion, caused, as we often see, by chewing sandy tobacco.

We sometimes meet with sensitiveness caused by fracture of a tooth; but the most prolific sources of sensitive dentine are the chemical corrodents. It is a general law of the human economy that when a solution of continuity is brought about in any part of the system, irritation, inflammation, pain, and soreness follow, and the teeth are no exception to this law.

Why is it then that we do not always find this exalted sensibility in the decayed teeth we are excavating? To this question we think we can give several plausible answers. The chemical agent corroding the tooth may have had a stronger affinity for the dentinal fibrils than it had for the dentine, and has devitalized them farther or deeper in than we are excavating; or it may be of such a character that it does not irritate or inflame them, also otherwise the corrodent may not be present at the time, nor for sometime previous, and consequently inflammation has subsided. Now the above are the reasons why we do not always find it. It next follows that we give the reasons why we do sometimes find it.

To this we answer whenever a chemical corrodent is in active operation upon the dentine, breaking down its structure, thereby exposing the nerve fibrils, yet not having sufficient affinity for the dentinal fibrils to devitalize them, but enough to irritate them and cause inflammation, then we have sensitive dentine, "so called."

Generally when a tooth receives a blow and is fractured, either the secretions of the mouth or the oxygen of the air coming in contact with the broken ends of the nerve fibrils inflame them, and make them very sensitive.

Whenever abrasion, either mechanical or chemical, on the grinding surface of the teeth goes on rapidly, removing the lime salts, and allowing the nerve fibrils to stand out flush with the surface on account of their exposure to the chemical and mechanical agents, they take on inflammation. As we have all seen, they get so sore sometimes as to prevent the person from chewing on them.

All these reasons may be more or less modified. For instance, patients having an inflammatory diathesis will suffer great pain from a lesion of any charactcr, also wounds received by them heal very slowly, and never by first intention. Whilst a person of a sound and healthy constitution, having great vitality, great powers of endurence and tenacity of life, suffer but very little even from severe wounds.

The structure of the teeth also modify the sensibility. Those of the greatest vasculariety being capable of taking on the highest sensibility; and this depends in a considerable measure upon the age of the patient. The younger the more vascularity, as they get older the tubuli become smaller by deposit of lime salts on their walls, which necessarily reduces the dentinal nerve fibrils.

Since we are continually meeting with this very troublesome thing, and it being the cause of the unpleasantness of many of our operations, also preventing us from receiving so full a practice, as it deters many persons from having their teeth filled; does it not seem strange that some of our scientific and skilful investigators have not found remedies suitable for each separate case. I have not found in our books any systematic or constitutional treatment. Here is a field for some of you to experiment in. It is in perfect accordance with reason to suppose that if the same systematic remedies are used here to allay inflammation, as are used to reduce it in other organs of the system, the results will be equally favorable, provided all the other conditions are observed necessary to favor the same. The most important of which would be to keep the chemical corrodent from the inflamed tissue.

We have asserted that sensitive dentine is a pathological phenomenon; if this is a fact, then it is subject or amendable to treatment. As practitioners of dental surgery, we ought to know the treatment indicated in each particular case, both locally and constitutionally.

To give you information or directions to meet all the cases you have to contend with, is more than your humble servant and co-laborer is qualified to do. But such information as I have picked up here and there, from the books and from living men, and what little I have noted in my short experience, I cheerfully give you, hoping it will lesson your labors, and afford some little relief to suffering humanity. It is only of a few local or topical remedies that we will speak. As before remarked, my experience is quite limited as to the use of the different remedies, but will endeavor to give it, and be as explicit as possible.

While speaking of individual remedies we will mention the particular cases wherein they are indicated or applicable. The shape and size of the cavity should always be taken into consideration; also the thickness of the septum of dentine between the decay and the pulp. We will begin with

*Tannic acid.* This is an astringent that has a strong affinity for albumen, gelatin and fibrin. The alcoholic solution should be used, as it will keep longer than any other. Its action on inflamed dentine is superficial and foild, and consequently cannot be relied upon for immediate relief or deep excavations; cannot be used with much benefit, except in connectien with temporary fillings.

*Creosote and carbolic acid.* We use carbolic acid because it is the stronger, more efficient, and has a less offensive odor. These are mild escharotics, uniting only with the animal portion of the tooth, and that very superficially, but ought to be and are universally used in every cavity just before filling, for their autiseptic effects. For their sedative effect on inflamed dentine they may be tried, and in many cases they will be found sufficient, but not effectual at all where there is a high degree of inflammation.

*Nitrate of silver.* This is a very energetic caustic, it seizes with avidity either the soft or bony tissues. It owes its caustic power to nascent, nitric acid. It has a strong affinity for albumen, uniting with it without undergoing decomposition. When first applied to albumen it turns it white, but as soon as the compound absorbs oxygen from the contiguous parts and the air, the compound is decomposed—the oxygen uniting with the silver forming oxyide of silver, and changes the color from white to black, while the nitric acid is set free and penetrates deeper, uniting with the soft and bony tissues present. It may be used in the solid state or a concentrated solution. It should never be used on a thin septum near the pulp, but is applicable to those acute sensitive points at the periphery of the dentine, where the dentinal fibrils terminate in papillæ or loops. There is not so much danger of this escharotic penetrating too deep as many operators suppose.

*Chloride of zinc.* This is a powerful caustic, very complicated in its action, as it produces antiseptic, disinfectant, and escharotic results. If applied in substance it produces considerable pain on account of its great affinity for water. Therefore it is better to use an etheral or chloroform solution, as they will supply it with water, while you derive some benefit from the local anasthetic effect from the ether or chloroform.

This agent must not be used near the pulps; but may be used in all the other parts of the cavity with marked advantage. You must give it a little time to take effect, but you must operate soon after, so that you will be done before reaction sets up.

The time of its action varies in different teeth. I tried chromic acid a few times. It gave so much pain I abondoned it altogether.

I have no doubt but the exalted sensibility of the dentine may be obtunded in some cases by the use of narcotics locally applied. I have not tried them sufficiently.

I had a very severe case not long since of sensitive dentine, that I succeeded satisfactorily with by the use of the narcotic spray. The case was that of a young lady of about twenty years. I am certain from the attempts I made at excavating prior to narcotizing that I never would have succeeded had I not used some agent to obtund the sensitiveness. The eavity was in the anterior proximal surface of the left central incisor.

After explaining to her the effect of the spray, I ordered it to be thrown on the tooth. When I stopped it, I quickly dried the parts that I might see where to cut, and then worked as rapidly as possible until the pain become too great. Then I would order the spray on again, and continued working and throwing the spray alternately until I had finished.

Surgical treatment of sensitive dentine consists in rapidly cutting away the inflamed surface. This answers if it is superficial. One author recommends rubbing a small burnisher on the sensitive part. I cannot indorse him.

